# Modulation of the antidepressant effects of ketamine by the mTORC1 inhibitor rapamycin

**DOI:** 10.1038/s41386-020-0644-9

**Published:** 2020-02-24

**Authors:** Chadi G. Abdallah, Lynnette A. Averill, Ralitza Gueorguieva, Selin Goktas, Prerana Purohit, Mohini Ranganathan, Mohamed Sherif, Kyung-Heup Ahn, Deepak Cyril D’Souza, Richard Formica, Steven M. Southwick, Ronald S. Duman, Gerard Sanacora, John H. Krystal

**Affiliations:** 1 National Center for PTSD – Clinical Neurosciences Division, US Department of Veterans Affairs, West Haven, CT USA; 2 Departments of Psychiatry, Neuroscience, and Psychology Yale University, New Haven, CT USA; 3 Department of Biostatistics, Yale University School of Public Health, New Haven, CT USA; 4 Department of Internal Medicine, Yale University School of Medicine, New Haven, CT USA

**Keywords:** Depression, Drug development

## Abstract

Twenty-four hours after administration, ketamine exerts rapid and robust antidepressant effects that are thought to be mediated by activation of the mechanistic target of rapamycin complex 1 (mTORC1). To test this hypothesis, depressed patients were pretreated with rapamycin, an mTORC1 inhibitor, prior to receiving ketamine. Twenty patients suffering a major depressive episode were randomized to pretreatment with oral rapamycin (6 mg) or placebo 2 h prior to the intravenous administration of ketamine 0.5 mg/kg in a double-blind cross-over design with treatment days separated by at least 2 weeks. Depression severity was assessed using Montgomery–Åsberg Depression Rating Scale (MADRS). Rapamycin pretreatment did not alter the antidepressant effects of ketamine at the 24-h timepoint. Over the subsequent 2-weeks, we found a significant treatment by time interaction (*F*_(8,245)_ = 2.02, *p* = 0.04), suggesting a prolongation of the antidepressant effects of ketamine by rapamycin. Two weeks following ketamine administration, we found higher response (41%) and remission rates (29%) following rapamycin + ketamine compared to placebo + ketamine (13%, *p* = 0.04, and 7%, *p* = 0.003, respectively). In summary, single dose rapamycin pretreatment failed to block the antidepressant effects of ketamine, but it prolonged ketamine’s antidepressant effects. This observation raises questions about the role of systemic vs. local blockade of mTORC1 in the antidepressant effects of ketamine, provides preliminary evidence that rapamycin may extend the benefits of ketamine, and thereby potentially sheds light on mechanisms that contribute to depression relapse after ketamine administration.

## Introduction

Ketamine is an N-methyl-d-aspartate receptor (NMDAR) antagonist that exerts rapid and robust antidepressant effects [1, 2]. The antidepressant effects may emerge within hours of a single dose, but without additional ketamine doses, relapse typically occurs in 3–14 days [3–5]. Ketamine and its metabolites are believed to exert antidepressant effects primarily by inducing a prefrontal glutamate neurotransmission surge leading to activation of synaptic α-amino-3-hydroxy-5-methyl-4-isoxazolepropionic acid glutamate receptors (AMPARs), which increases brain-derived neurotrophic factor (BDNF) levels, enhances stimulation of TrkB receptors, activates the mechanistic target of rapamycin complex 1 (mTORC1), and produces synaptogenesis [6–9]. Several preclinical studies have shown that ketamine administration increases mTORC1 signaling [10–13], but there are non-replications of this finding [14, 15]. Most importantly, a single infusion of rapamycin into the medial prefrontal cortex (PFC) prior to ketamine injection in rodents was reported to block the neuroplasticity and antidepressant-like effects of ketamine [10, 16].

The current study was designed to test the hypothesis that the antidepressant effects of ketamine are mediated by activation of mTORC1 by evaluating whether the antidepressant effects of ketamine, observed in depressed patients 24 h after administration, are blocked by pretreatment with the mTORC1 inhibitor, rapamycin. Following an experimental paradigm derived from animal research [10, 16], we aimed to demonstrate in patients the observation that rapamycin blocks the antidepressant-like effects of ketamine [10, 16].

In initial test of the mTORC1 hypothesis of ketamine effects in humans, we were aware of two important concerns. First, we wished to test a rapamycin dose that would be tolerable to research subjects, raising the possibility that underdosing of rapamycin might affect findings. Second, rapamycin has powerful anti-inflammatory effects that might directly produce antidepressant effects [17–21] that might confound interpretation of the study findings. Because the anti-inflammatory effects of rapamycin might augment those of ketamine [22] and enhance treatment efficacy, we followed patients for 2 weeks after each ketamine dose.

Using a randomized placebo-controlled cross-over design, rapamycin was administered as a single 6 mg dose prior to ketamine infusion. In several species, preclinical studies have shown that rapamycin crosses the blood brain barrier, as measured by rapamycin levels in the cerebrospinal fluid and brain tissues, or by the inhibition of brain mTORC1 signaling [23–26]. Moreover, within 2 h following peripheral rapamycin administration, one study reported decreased phosphorylation of S6 ribosomal protein in brain tissues—a pharmacodynamic readout of mTORC1 inhibition [15]. Furthermore, the immunosuppressive effect of rapamycin is an mTORC1-dependent process [27] and rapamycin was shown at therapeutic doses in humans to cross the brain blood barrier and to reduce the phosphorylation of S6 ribosomal protein in brain tissue [28, 29]. Therefore, the rapamycin dose and timing were selected based on the drug pharmacokinetics to ensure, at the time of ketamine administration, blood concentration of 5–20 ng/mL, a level that exhibits potent immunosuppression [29]. Consistent with the hypothesized mechanism of action of ketamine, we predicted that rapamycin would reduce the antidepressant effects of ketamine.

## Materials and methods

### Study design

All study procedures were approved by institution review boards at Yale and Connecticut Veteran Affairs Hospital, and all participants completed an informed consent process prior to enrollment (*ClinicalTrials.gov: NCT02487485*). A Data and Safety Monitoring Board (DSMB) oversaw the study protocol and monitored the study progress. The clinical trial included two study phases (I and II).

In phase I (September–December 2015), three participants received open-label oral rapamycin (a.k.a. sirolimus) followed 2 h later by open-label intravenous ketamine. Participants remained on the research unit for at least 10 h following the administration of rapamycin and were discharged upon clearance by the covering physician. The aim of phase I was to qualitatively assess the safety and feasibility of the co-administration of rapamycin and ketamine. The three participants tolerated the rapamycin + ketamine combination well, without any residual symptoms or unexpected adverse events. Therefore, the study proceeded to phase II.

In phase II (June 2016–February 2018), 23 participants were randomized to first receive either rapamycin or placebo, followed 2 h later by open-label ketamine (see CONSORT Diagram in Supplementary material). Phase II was a double-blind, placebo-controlled, cross-over design with at least 2 weeks between Infusion 1 (i.e., 1st treatment day) and Infusion 2 (i.e., 2nd treatment day). Randomization assignments were generated and assigned by the research pharmacist according to a block randomization with block size of six. Individuals, clinicians, investigators, and research staff were blinded to randomization assignments. Depression severity no less than 80% of baseline was required prior to proceeding with Infusion 2. If the participant did not meet this severity criteria, the infusion 2 was rescheduled until the following week. Participants who received placebo on Infusion 1 received rapamycin on Infusion 2, and vice-versa. Both study phases used an oral single-dose of 6 mg rapamycin in liquid form, diluted in orange juice to maintain the blinding, and ketamine 0.5 mg/kg intravenously infused over 40 min. Ketamine administration and monitoring was comparable to previous studies [1, 2, 30]. Preplanned interim analysis of the first six subjects confirmed that the rapamycin level is reaching therapeutic levels. Hence, the study continued randomization without rapamycin dose adjustments. Participants were assessed up to 2 weeks following each Infusion.

### Study criteria

The study enrolled subjects between the age of 21 and 65 years, recruited through advertisement and referrals from outpatient clinics. Participants were (1) diagnosed with current major depressive episode, (2) had a history of non-response to at least one adequate antidepressant trial, (3) were unmedicated or on a stable antidepressant or psychotherapy for at least 4 weeks prior to randomization, then during the study, (4) had a MADRS ≥ 18 prior to randomization, (5) females were not pregnant or breastfeeding and were on a medically acceptable contraceptive method, (6) were able to read, write, and provide written informed consent, (7) did not have psychotic disorder or features, or current manic or mixed episodes, (8) did not have an unstable medical condition, (9) did not require prohibited medications (see Table S1 in Supplementary material), (10) did not have urine drug screen positive for cannabis, phencyclidine, cocaine, or barbiturates, (11) had no substance dependence within 3 months, (12) had no known sensitivity to rapamycin, ketamine, or heparin as reported by the subjects, and (13) had resting blood pressure higher than 85/55 and lower than 150/95 mmHg, and heart rate higher than 45/min and lower than 100/min.

### Outcomes

Assessment measures included: (1) the Mini International Neuropsychiatric Interview (MINI) to determine the diagnosis, (2) Montgomery Åsberg Depression Rating Scale (MADRS) as primary outcome of depression severity, (3) Quick Inventory of Depressive Symptoms Self-Report (QIDS-SR) and Hamilton Anxiety Rating Scale (HAMA) as secondary measures of depression and anxiety severity, respectively, (4) Clinician Administered Dissociative States Scale (CADSS) and Positive and Negative Symptom Scale (PANSS), as safety measures of the psychotomimetic effects of ketamine, (5) rapamycin level immediately before starting ketamine and ~4 h later, (6) ketamine level before the end of each infusion and (7) high-sensitivity C-reactive protein (CRP) and erythrocyte sedimentation rate (ESR) prior to randomization to examine whether baseline inflammatory markers affect the antidepressant response to ketamine.

The study a priori primary outcome was MADRS. Response was defined as 50% improvement, and remission was defined as MADRS < 10 [31]. MADRS scores were measured immediately prior to rapamycin and placebo administration, and after starting ketamine infusion at 1, 2, 4 h, 3 days, 5 days, 1 week, and 2 weeks.

### Statistics

Descriptive statistics (means, standard deviations, and frequencies) were calculated prior to statistical analysis. Data distributions were checked using normal probability plots. Outcome variables were analyzed using mixed models with fixed effects of treatment (rapamycin vs. placebo), time (appropriate time points during Infusions 1 and 2), the interaction between treatment and time, and order (placebo first vs. rapamycin first). The best-fitting variance–covariance structure for each model was selected based on the Schwartz’ Bayesian Information criterion. Interactions between order and the other factors were checked for significance but not included in the final models for parsimony. Similarly, the effects of the variables CRP and ESR (log-transformed) were checked for significance, but since these were non-significant, they were removed from the final models. Post-hoc tests were used to interpret significant effects in the models: comparisons of treatment conditions by time-point for significant rapamycin by time interactions, and pairwise comparisons of time points for significant main effects of time. Least-square means and standard errors by treatment and by time were used for visualization of results. The response and remission rates were compared between treatments using McNemar test. Effect size (Cohen’s *d*′) was calculated as the mean of the within-subject difference over its standard deviation. Correlation analyses explored the relationship between rapamycin level and improvement in depression severity. All tests are two-tailed with significance set at *p* < 0.05.

The sample size was targeted based on feasibility within the 3-year funding available for this discovery phase project. Initially, we aimed to randomize 30 subjects in 3 years. The targeted sample provides 80% power for detecting ketamine–rapamycin differences of moderate size (Cohen’s *d*′ = 0.55), assuming a two-tailed alpha = 0.05. However, we had a 1-year delay in starting randomization due to the addition of Phase 1 and the need for an investigational new drug exemption, both of which were requested by the institution review board. Thus, we were able to randomize a total of 23 patients in 2 years, 20 of them were included in the analysis. With 20 individuals, under the same assumptions, the detectable effect size is *d*′ = 0.68. Following randomization, one participant was excluded from the primary analysis due to receiving high dose hydrocortisone the night before randomization and the DSMB was informed accordingly. The decision to exclude the participant was made prior to compiling and unblinding the study data. However, for full transparency, a secondary analysis including this participant was conducted and reported in the Supplementary material. The results were found to be comparable to those of the primary analysis.

## Results

### Participants

As detailed in the CONSORT Flow Diagram (see Supplementary material), 23 of the 57 assessed for eligibility were randomized and 20 participants were included in the analysis (2 subjects did not meet study criteria the morning of the first treatment day, and 1 subject received high dose hydrocortisone the night of the treatment day). As detailed in Table 1, the 20 participants were 8 men and 12 women, with mean (±SEM) age = 42.8 (±2.8) years, BMI = 27.2 (±1.3) kg/m^2^, CRP = 2.4 (±0.8) mg/L, ESR = 11.5 (±2.3) mm/h, pre-infusion rapamycin = 26.5 (±2.4) ng/mL, and 4 h post-infusion rapamycin = 9.9 (±1.0) ng/mL. There was no difference in ketamine level between study arms (mean ± SEM placebo = 125 ± 13 ng/mL vs. rapamycin = 115 ± 16 ng/mL, df = 17, *p* = 0.63). Recruitment and follow-up were conducted between September 2015 and February 2018.

**Table 1:** Demographics and clinical characteristics.

	Patients (*n* = 20)
Age (mean ± SEM)	42.8 ± 2.8 years
Women	12
Race (white)	15
Education (mean ± SEM)	8.4 ± 0.3 years
BMI (mean ± SEM)	27.2 ± 1.3 kg/m^2^
CRP (mean ± SEM)	2.4 ± 0.8 mg/L
ESR (mean ± SEM)	11.5 ± 2.3 mm/h
T-0 h Rapamycin (mean ± SEM)	26.5 (±2.4) ng/mL
T-4 h Rapamycin (mean ± SEM)	9.9 (±1.0) ng/mL
Concomitant medications	None (*N* = 1)
	SRI (*N* = 14)
	Mood stabilizer or SGA (*N* = 3)
	Other antidepressants (*N* = 5)
	Stimulants (*N* = 2)
	Sedatives (*N* = 7)
	Other psychotropics (*N* = 3)
	Non-psychotropics (*N* = 10)^a^
Psychiatric hospitalization history	8
Family psychiatric history	12
Treatment failures (mean ± SEM)	5.1 (±0.9)

*SEM* standard error of means, *BMI* body mass index, *CRP* C reactive protein, *ESR* erythrocyte sedimentation rate, T-0 h immediately prior to ketamine infusion, T-4 h four hours post infusion, *SRI* serotonin reuptake inhibitor, *SGA* second generation antipsychotics.

^a^Non-psychotropics were primarily for diabetes, hypertension, GERD, or hypothyroidism.

### Treatment effects on MADRS

**Fig. 1:**
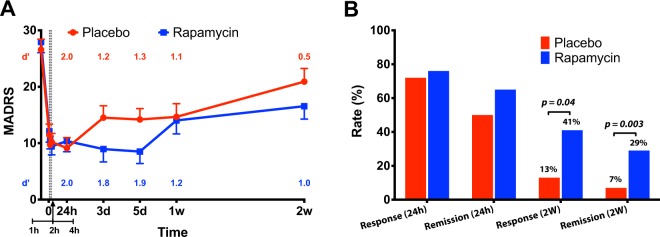
The study drug effect on Montgomery Åsberg Depression Rating Scale (MADRS). **a** There is a significant main effect of time (*F*_(8,245)_ = 43.5, *p* < 0.0001), demonstrating significant decrease in MADRS scores from baseline. There is also significant interaction between treatment and time (*F*_(8,245)_ = 2.0, *p* = 0.04), with overall reduction in depression scores following treatment with rapamycin + ketamine (rapamycin; blue line), compared to post placebo + ketamine (placebo; red line). Primary outcome time points included: before infusion, and 1, 2, 4, 24 h, 3 days, 5 days, 1 week, and 2 weeks after infusion. Dashed lines refer to assessments at 1, 2, and 4 h. **b** Response and remission rates following treatment with rapamycin + ketamine (rapamycin; blue), compared to post placebo + ketamine (placebo; red). *Notes*: Error bars are standard errors of mean (SEM); *d*′ = Cohen’s *d*′ effect size compared to pretreatment MADRS scores.

**Fig. 2:**
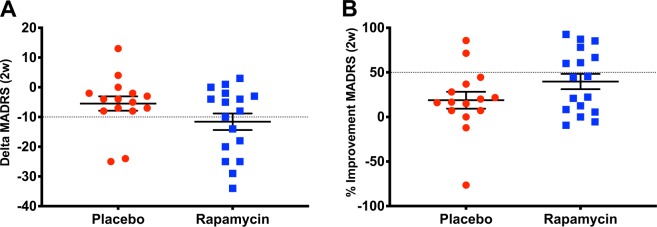
A scatter plot of the changes in Montgomery Åsberg Depression Rating Scale (MADRS) at 2 weeks. **a** Delta MADRS is the score at 2 weeks minus pre-treatment. **b** Percent improvement in MADRS scores at 2 weeks. The dotted line separate the responders from non-responders.

MADRS was selected a priori as the primary outcome. There was a statistically significant interaction between treatment and time (*F*_(8,245)_ = 2.0, *p* = 0.04, Fig. 1a), with significant differences between rapamycin and placebo at day 3 (*p* = 0.04), and at day 5 (*p* = 0.02). There was also a significant main effect of time (*F*_(8,245)_ = 43.5, *p* < 0.0001), demonstrating significant decrease in MADRS scores from baseline, with the highest numerical mean difference achieved at 24 h (17.5 ± 1.4) and then gradually reduced until 2 weeks (8.5 ± 1.7). However, the mean MADRS scores at 2 weeks remained significantly lower than baseline following both placebo (*Cohen’s d‘* = 0.5; mean difference (±SEM) = 5.7 (±2.5), *t*_(245)_ = 2.3, *p* = 0.02) and rapamycin treatments (*Cohen’s d‘* = 1.0; mean difference (±SEM) = 11.4 (±2.4), *t*_(245)_ = 4.7, *p* < 0.0001; Fig. 1a). There was no significant main effect of treatment (*F*_(8,245)_ = 1.4, *p* = 0.24) and the effects of the variables CRP and ESR were non-significant (*p* > 0.1). At 24 h, the response (72%; *N* = 13) and remission (50%; *N* = 9) rates following placebo were comparable to post rapamycin treatment (76%; *N* = 13 and 65%; *N* = 11, *p* > 0.05). In contrast, at 2 weeks, the response (13%; *N* = 2) and remission (7%; *N* = 1) rates following placebo were lower than response (41%; *N* = 7; Figs. 1b and 2) and remission (29%; *N* = 5) rates following rapamycin treatment (*p* = 0.04 and *p* = 0.003, respectively).

### Treatment effects on QIDS-SR and HAMA

There was a significant main effect of time on QIDS-SR (*F*_(8,236)_ = 7.1, *p* < 0.0001; Fig. S1), demonstrating significant decrease in QIDS-SR scores from baseline, with the highest numerical mean difference achieved at day 3 (5.2 ± 1.2) and then gradually reduced until 2 weeks (2.1 ± 1.1). The mean QIDS-SR scores at 2 weeks remained significantly lower than baseline following rapamycin treatment (*Cohen’s d‘* = 0.5; mean difference (±SEM) = 3.5 (±1.5), *t*_(236)_ = 2.4, *p* = 0.02), but not following placebo (*Cohen’s d‘* = 0.1; mean difference (±SEM) = 0.7 (±1.5), *t*_(236)_ = 0.5, *p* = 0.64; Fig. S1). There was no significant main effect of treatment (*F*_(8,236)_ = 0.3, *p* = 0.57) or interaction between treatment and time (*F*_(8,236)_ = 0.5, *p* = 0.87). At 24 h, there were no significant differences (*p* > 0.9) in the rate of patients who showed 50% improvement on QIDS-SR (placebo + ketamine: 44% vs. rapamycin + ketamine: 47%). However, there was a significantly higher rate of patients (*p* < 0.05) who showed 50% improvement on QIDS-SR at 2 weeks following rapamycin + ketamine (38%), compared to placebo + ketamine (8%).

There was a significant main effect of time on HAMA (*F*_(4,141)_ = 31.2, *p* < 0.0001), demonstrating significant decrease in HAMA scores from baseline, with the highest numerical mean difference achieved at 4 h (9.9 ± 1.0) and then gradually reduced until 2 weeks (3.0 ± 1.2). The mean HAMA scores at 2 weeks was not significantly different compared to baseline following both placebo (*Cohen’s d‘* = 0.4; mean difference (±SEM) = 3.0 (±1.8), *t*_(141)_ = 1.6, *p* = 0.11) and rapamycin treatments (*Cohen’s d‘* = 0.4; mean difference (±SEM) = 3.1 (±1.7), *t*_(141)_ = 1.9, *p* = 0.07). There was no significant main effect of treatment (*F*_(1,141)_ = 0.2, *p* = 0.68) or interaction between treatment and time (*F*_(4,141)_ = 0.7, *p* = 0.63).

### Adverse effects

**Fig. 3:**
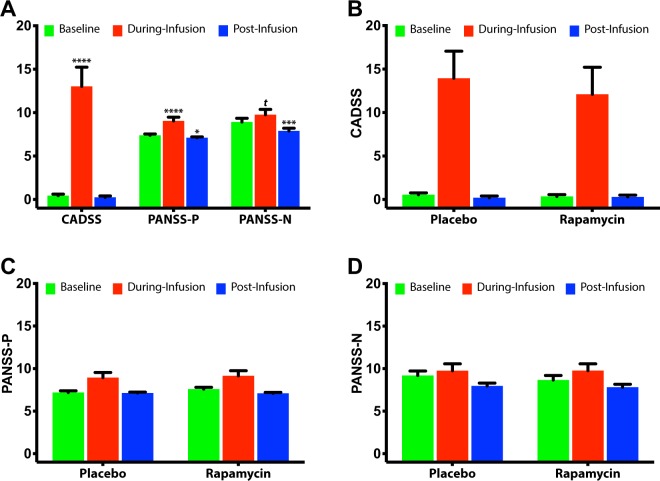
The study drug effect on Clinician Administered Dissociative States Scale (CADSS), and Positive and Negative Symptom Scale (PANSS) positive (PANSS-P) and negative symptoms (PANSS-N). Error bars are standard errors of mean (SEM); **a** Comparisons to pretreatment scores are marked with **** for *p* ≤ 0.0001, *** for *p* ≤ 0.001, * for *p* ≤ 0.05, and *t* for *p* = 0.10. **b**–**d** There were no treatment effects or treatment by time interactions.

There was a significant main effect of time on CADSS (*F*_(2,95)_ = 18.9, *p* < 0.0001), demonstrating significant increase in CADSS scores during infusion, which returned to baseline 2 h post infusion (Fig. 3a). There was no significant main effect of treatment (*F*_(2,95)_ = 0.2, *p* = 0.67) or interaction between treatment and time (*F*_(2,95)_ = 0.5, *p* = 0.60; Fig. 3b).

There was a significant main effect of time on PANSS-positive (*F*_(2,82)_ = 11.3, *p* < 0.0001), demonstrating significant increase in PANSS-positive scores during infusion, with significant reduction 2 h post infusion (Fig. 3a). There was no significant main effect of treatment (*F*_(2,82)_ = 0.3, *p* = 0.57) or interaction between treatment and time (*F*_(2,82)_ = 1.9, *p* = 0.15; Fig. 3c). There was a significant main effect of time on PANSS-negative (*F*_(2,82)_ = 11.9, *p* < 0.0001), demonstrating significant reduction 2 h post infusion (Fig. 3a). There was no significant main effect of treatment (*F*_(2,82)_ = 0.2, *p* = 0.68) or interaction between treatment and time (*F*_(2,82)_ = 0.3, *p* = 0.73; Fig. 3d).

Participants in Phase 1 tolerated the combination treatment with no serious or unexpected adverse events. The study drug effects were clinically comparable to previous ketamine studies and there was no need for the extended monitoring of 10 h. Therefore, we proceeded with the second phase double-blind randomization and participants were discharged with transportation to home after medical clearance and completion of the last assessment on each treatment day. The adverse events during Phase 2 are reported in Table S2. There were no serious adverse events. New onset adverse events were mostly mild and transient. There were no reports of persistent adverse events. The most frequent adverse events were fatigue, headaches, nausea, and pain. A total of 37 events were reported, 21 of which were reported by four participants.

## Discussion

This study yielded two surprising, but potentially important, clinical observations. First, this study failed to validate the prediction from preclinical studies [10, 16], in that rapamycin pretreatment did not reduce the acute antidepressant effects of ketamine at 24 h following treatment. At 24 h, the depression scores and response rates were highly comparable between study arms. Second, rapamycin pretreatment increased the response and remission rates at 2 weeks (Fig. 1b), suggesting that this treatment approach may prolong the antidepressant effects of ketamine. This conclusion is supported by the statistically significant drug by treatment interaction effect on the primary outcome MADRS, showing overall larger reduction in depression scores following rapamycin pretreatment (Fig. 1a). Additionally, the Cohen’s *d*′ effect size at 2 weeks post rapamycin was 1.0, compared to 0.5 following placebo pretreatment (Fig. 1a). Moreover, the reduction in QIDS-SR scores (secondary outcome) at 2 weeks were significant following rapamycin, but not placebo pretreatment. As well as the response rate using QIDS-SR which was significantly higher at 2 weeks following rapamycin treatment. However, it is important to note the lack of significant difference at 1 week which may indicate a fluctuating course, or it may be related to the relatively small sample size.

While preliminary in nature, the unanticipated finding of prolonged response is highly important considering the urgent need for treatment approaches to prolong the antidepressant effects of ketamine and other rapid-acting antidepressants. While infusions of ketamine 2–3 times per week have been shown to afford clinical benefit during an induction period, less frequent administration is preferable to reduce the patient burden, adverse events, and drug abuse liability. Additionally, rapamycin pretreatment appears to have no effects on the blood level, the anxiolytic or the psychotomimetic effects of ketamine. This suggests that the prolongation of the antidepressant effects was not a consequence of increased blood levels of ketamine or changes in the subjective response to ketamine. Overall, rapamycin and ketamine were well tolerated with no serious adverse events.

### Why did peripherally administered rapamycin fail to block the antidepressant effects of ketamine?

In humans, we are not able to administer rapamycin intracortically to fully parallel the preclinical reports. Further, we limited exposure to rapamycin to a loading immunosuppressant dose, which was selected on the basis of being the highest dose one could administer without exposing subjects to a risk of side effects associated with higher doses [32]. However, we believed that it was important to test whether systemic mTORC1 inhibition blocks the antidepressant effects of ketamine in humans because: (1) The immunosuppressant effects of rapamycin are mTORC1-dependent [27]. (2) There are preclinical and clinical reports providing evidence that peripherally administered rapamycin crosses the blood brain barrier and actively inhibit brain mTORC1 signaling [23–26, 28, 29]. (3) Acute single dose of rapamycin administered peripherally was shown to inhibit mTORC1 in the brain within 2 h of administration in rodents [15]. This body of information justified testing whether an immunosuppressant dose of rapamycin would be sufficient to block the antidepressant effects of ketamine in depressed patients. The current study findings rejected this hypothesis. However, it remains plausible that higher oral doses of rapamycin or intracortical local administration of rapamycin is required to inhibit the antidepressant effects of ketamine. Indeed, there are preclinical studies demonstrating inhibition of the ketamine effects following intracortical, but not peripheral administration of rapamycin (e.g., refs. [10, 15]). Moreover, rodent and human evidence show region-specific neuroplasticity effects of ketamine consistent with increased synaptic connectivity in the PFC and hippocampus, but decreased synaptic connectivity in the nucleus accumbens (NAc) [33, 34]. The opposing changes in neuroplasticity were independently related to successful ketamine treatment [33]. Therefore, the failure of systemic rapamycin may be due to comparable reduction in synaptic formation in both the PFC and NAc. This hypothesis is particularly relevant considering recent evidence showing plasticity-independent acute antidepressant effects of ketamine [35].

While the rapamycin dose and route of administration provides a putative explanation for the discrepancy between human and animal data, other alternative possibilities should be considered. In particular, the validity of the preclinical model of depression that gave rise to the mTORC1 hypothesis. Unfortunately, results from preclinical models do not always translate into evidence in human clinical trials. At least partially, this may be due to the complexity of depression, which is an episodic illness with some genetic component, compared to the rodent models which are mostly stress related. In light of the current results, the route of administration and the validity of related basic models should be carefully considered in the ongoing effort to target mTORC1 for novel treatment development, especially considering that antidepressant medications are often administered peripherally. Finally, recent studies have shown plasticity-independent acute antidepressant effects [35] and other studies suggested that some of the clinical benefit of ketamine can be attributed to non-specific effects of the treatment [36]. Thus, rapamycin would not necessarily inhibit these plasticity-independent and off-target effects.

### Why are the antidepressant effects of ketamine transient and why are these effects prolonged by rapamycin?

One possibility suggested by this study is that ketamine treats depression without resolving underlying processes, such as inflammation, that produce synaptic elimination and undermine the antidepressant effects of ketamine. This hypothesis presumes that the expression of the antidepressant effects of ketamine depends upon sustaining the newly made synapses [6, 37]. The anti-inflammatory effects of rapamycin may protect these synapses and thereby extend the antidepressant effects of ketamine.

A second possibility is that rapamycin prolongs the antidepressant effects of ketamine by enhancing autophagy. Neuronal plasticity is thought to be critical in the pathology and treatment of depression, particularly in the mechanisms of rapid acting antidepressants [7]. Autophagy, which is regulated by mTORC1, plays an essential role in normal cellular plasticity, by degrading and recycling toxic or dysfunctional cellular components. Recent evidence implicates autophagy in the mechanisms of antidepressants [38]. Moreover, rapamycin and other autophagy enhancers were previously shown to exert antidepressant-like effects in preclinical studies [39, 40]; although the effects of rapamycin were evident following repeated, but not acute administration [15, 39, 40].

## Limitations and strengths

As a first-in-humans study, the study sample was based on feasibility and funding availability rather than a priori knowledge of effect size. Therefore, the lack of treatment by time interaction for QIDS-SR may be the result of insufficient power to demonstrate a significant effect on this self-report measure of depression severity, which tends to have higher variability. Consistent with this possibility, the QIDS-SR Cohen’s *d*′ effect size at 2 weeks post rapamycin was 0.5, compared to only 0.1 following placebo treatment (Fig. S1). Moreover, the response rate using QIDS-SR was also significantly higher at 2 weeks following rapamycin + ketamine compared to placebo + ketamine. Based on our observation in the first phase of the study, we did not ask the participants to guess their treatment in Phase 2, as it was evident that the patients were unable to identify a rapamycin taste in the juice and the side effects were comparable to those seen in previous ketamine studies. Future studies may consider the benefit of adding an objective measure to determine the efficacy of the blinding. Additionally, we did not examine whether ketamine metabolites (e.g., hydroxynorketamine) were affected by rapamycin, subsequently leading to the prolonged antidepressant effects. However, both ketamine and rapamycin have been in clinical use for a long time with no known reported or theoretical metabolic interactions; particularly because rapamycin is metabolized by CYP3A4, while ketamine is primarily metabolized through CYP2B6. Finally, although rapamycin reached therapeutic levels, future studies are encouraged to investigate cerebral spinal fluid levels of rapamycin to determine whether its relapse prevention effects are through central or peripheral mechanisms.

A main strength of the study is the attempt to investigate an essential mechanistic pathway, that has been so far implicated in the pathology and treatment of depression based primarily on preclinical evidence. Other strengths of the study include a double-blind randomized placebo-controlled cross-over design. Here, it is important to underscore that, to our knowledge, the current study is one of the largest cross-over ketamine studies in depression [2, 30, 41, 42]. In contrast to parallel groups design [1, 43, 44], cross-over ketamine studies required smaller samples because of the robust effects of ketamine and the rapid relapse, leading to increased statistical power to detect significant within subject differences. In fact, for the first 12 years following the discovery of the antidepressant effects of ketamine all controlled studies were mainly cross-over and smaller in size compared to the current study [2, 30, 41, 42]. However, similar to the ketamine discovery, it remains critical that the current rapamycin + ketamine findings are replicated in future independent trials.

Finally, the fact that the immune system is involved in both depression pathology as well as in resilience and depression recovery [45] creates a major challenge in the field, emphasizing the need to target a “sweet spot” that will oppose the negative effects of inflammation while avoiding the inhibition of its neuroregulatory function [45]. Therefore, an essential strength of the study is the use of combined therapy, instead of monotherapy or add-on approaches that were used in the past [19]. If successfully developed as one drug administration every 7–14 days, combined therapy will overcome many of the shortcomings of anti-inflammatory monotherapy/add-on approaches, particularly that the effect appears to be independent of pretreatment exaggerated inflammatory state (i.e., no CRP or ESR effects), which was suggested to be necessary for successful monotherapy/add-on approaches [46].

## Conclusion

The administration of a single dose of rapamycin, reaching blood levels known to induce potent immunosuppression, does not inhibit the rapid acting antidepressant effects of ketamine at 24 h post treatment. Intriguingly, the immunosuppressant rapamycin prolonged the antidepressant effects of ketamine, and increased the response and remission rates at 2 weeks following treatment. This preliminary evidence requires replication in future studies. To date, preclinical and clinical studies based on the synaptic model of depression have largely focused on the transient alteration in synaptic density. Future studies providing greater insight into the mechanisms of synaptic density stabilization and approaches to target autophagy may provide novel target for drug development and could ultimately lead to depression cure rather than treatment.

## Funding and disclosure

Funding and research support were provided by Gustavus and Louise Pfeiffer Research Foundation, NIMH (K23MH101498), and the VA National Center for PTSD, the CT Department of Mental Health and Addiction Services and Yale-New Haven Health. Dr. Abdallah has served as a consultant, speaker and/or on advisory boards for Genentech, Janssen, Psilocybin Labs, Lundbeck and FSV7, and editor of *Chronic Stress* for Sage Publications, Inc.; Filed a patent for using mTORC1 inhibitors to augment the effects of antidepressants (filed on August 20, 2018). JHK is a consultant for AbbVie, Inc., Amgen, Astellas Pharma Global Development, Inc., AstraZeneca Pharmaceuticals, Biomedisyn Corporation, Bristol-Myers Squibb, Eli Lilly and Company, Euthymics Bioscience, Inc., Neurovance, Inc., FORUM Pharmaceuticals, Janssen Research & Development, Lundbeck Research USA, Novartis Pharma AG, Otsuka America Pharmaceutical, Inc., Sage Therapeutics, Inc., Sunovion Pharmaceuticals, Inc., and Takeda Industries; is on the Scientific Advisory Board for Lohocla Research Corporation, Mnemosyne Pharmaceuticals, Inc., Naurex, Inc., and Pfizer; is a stockholder in Biohaven Pharmaceuticals; holds stock options in Mnemosyne Pharmaceuticals, Inc.; holds patents for Dopamine and Noradrenergic Reuptake Inhibitors in Treatment of Schizophrenia, U.S. Patent No. 5,447,948 (issued September 5, 1995), and Glutamate Modulating Agents in the Treatment of Mental Disorders, U.S. Patent No. 8,778,979 (issued July 15, 2014); and filed a patent for Intranasal Administration of Ketamine to Treat Depression—U.S. Application No. 14/197,767 (filed on March 5, 2014); U.S. application or Patent Cooperation Treaty international application No. 14/306,382 (filed on June 17, 2014). Filed a patent for using mTORC1 inhibitors to augment the effects of antidepressants (filed on August 20, 2018). RG discloses consulting fees for Palo Alto Health Sciences, Knopp Biosciences and Mathematica Policy Research, royalties from book “Statistical Methods in Psychiatry and Related Fields” published by CRC Press, and a provisional patent submission by Yale University: Chekroud, AM., Gueorguieva, R., & Krystal, JH. “Treatment Selection for Major Depressive Disorder” [filing date 3rd June 2016, USPTO docket number Y0087.70116US00]. GS has received consulting fees from Alkermes, Allergan, AstraZeneca, Avanier Pharmaceuticals, Axsome Therapeutics, Biohaven Pharmaceuticals, Boehringer Ingelheim, Bristol-Myers Squibb, Hoffmann–La Roche, Intra-Cellular Therapies, Janssen, Merck, Minerva Neurosciences, Naurex, Navitor Pharmaceuticals, Novartis, Noven Pharmaceuticals, Otsuka, Perception Neuroscience, Praxis Therapeutics, Sage Pharmaceuticals, Servier Pharmaceuticals, Taisho Pharmaceuticals, Teva, Valeant, and Vistagen Therapeutics. He has also received research contracts from AstraZeneca, Bristol-Myers Squibb, Eli Lilly, Johnson & Johnson, Hoffmann–La Roche, Merck, Naurex, and Servier Pharmaceuticals. No-cost medication was provided to GS for an NIH-sponsored study by Sanofi-Aventis. In addition, he holds shares in Biohaven Pharmaceuticals Holding Company and is a co-inventor on the patent “Glutamate agents in the treatment of mental disorders” (patent 8778979). RF is a consultant for Veloxis Pharmaceutical and Norvatis Pharmaceuticals. In addition, he is secretary of the American Society of Transplantation. DCD receives research support administered through Yale University School of Medicine currently from Takeda, and in the past 3 years from INSYS Therapeutics. MR has in the past 3 years, or currently receives, research grant support administered through Yale University School of Medicine from INSYS Therapeutics. All other co-authors declare no conflict of interest.

## Supplementary information

Suplemental Information

## References

[1] Murrough JW, Iosifescu DV, Chang LC, Al Jurdi RK, Green CE, Perez AM (2013). Antidepressant efficacy of ketamine in treatment-resistant major depression: a two-site randomized controlled trial. Am J Psychiatry.

[2] Zarate CA, Singh JB, Carlson PJ, Brutsche NE, Ameli R, Luckenbaugh DA (2006). A randomized trial of an N-methyl-d-aspartate antagonist in treatment-resistant major depression. Arch Gen Psychiatry.

[3] Aan Het Rot M, Zarate CA, Charney DS, Mathew SJ (2012). Ketamine for depression: where do we go from here?. Biol Psychiatry.

[4] Abdallah CG, Averill LA, Krystal JH (2015). Ketamine as a promising prototype for a new generation of rapid-acting antidepressants. Ann NY Acad Sci.

[5] Romeo B, Choucha W, Fossati P, Rotge JY (2015). Meta-analysis of short- and mid-term efficacy of ketamine in unipolar and bipolar depression. Psychiatry Res..

[6] Abdallah CG, Sanacora G, Duman RS, Krystal JH (2018). The neurobiology of depression, ketamine and rapid-acting antidepressants: is it glutamate inhibition or activation?. Pharm Ther..

[7] Gould TD, Zarate CA, Thompson SM (2019). Molecular pharmacology and neurobiology of rapid-acting antidepressants. Annu Rev Pharm Toxicol.

[8] Murrough JW, Abdallah CG, Mathew SJ (2017). Targeting glutamate signalling in depression: progress and prospects. Nat Rev Drug Discov.

[9] Ignacio ZM, Reus GZ, Arent CO, Abelaira HM, Pitcher MR, Quevedo J (2016). New perspectives on the involvement of mTOR in depression as well as in the action of antidepressant drugs. Br J Clin Pharmacol.

[10] Li N, Lee B, Liu RJ, Banasr M, Dwyer JM, Iwata M (2010). mTOR-dependent synapse formation underlies the rapid antidepressant effects of NMDA antagonists. Science.

[11] Zhou W, Wang N, Yang C, Li XM, Zhou ZQ, Yang JJ (2014). Ketamine-induced antidepressant effects are associated with AMPA receptors-mediated upregulation of mTOR and BDNF in rat hippocampus and prefrontal cortex. Eur Psychiatry.

[12] Yang C, Hu YM, Zhou ZQ, Zhang GF, Yang JJ (2013). Acute administration of ketamine in rats increases hippocampal BDNF and mTOR levels during forced swimming test. Ups J Med Sci.

[13] Harraz MM, Tyagi R, Cortes P, Snyder SH (2016). Antidepressant action of ketamine via mTOR is mediated by inhibition of nitrergic Rheb degradation. Mol Psychiatry.

[14] Popp S, Behl B, Joshi JJ, Lanz TA, Spedding M, Schenker E, et al. In search of the mechanisms of ketamine’s antidepressant effects: How robust is the evidence behind the mTor activation hypothesis. F1000Res. 2016;5:634. 10.12688/f1000research.8236.1.

[15] Autry AE, Adachi M, Nosyreva E, Na ES, Los MF, Cheng PF (2011). NMDA receptor blockade at rest triggers rapid behavioural antidepressant responses. Nature.

[16] Li N, Liu RJ, Dwyer JM, Banasr M, Lee B, Son H (2011). Glutamate N-methyl-d-aspartate receptor antagonists rapidly reverse behavioral and synaptic deficits caused by chronic stress exposure. Biol Psychiatry.

[17] Haroon E, Miller AH, Sanacora G (2017). Inflammation, glutamate, and glia: a trio of trouble in mood disorders. Neuropsychopharmacology.

[18] Miller AH (2013). Conceptual confluence: the kynurenine pathway as a common target for ketamine and the convergence of the inflammation and glutamate hypotheses of depression. Neuropsychopharmacology.

[19] Kohler O, Benros ME, Nordentoft M, Farkouh ME, Iyengar RL, Mors O (2014). Effect of anti-inflammatory treatment on depression, depressive symptoms, and adverse effects: a systematic review and meta-analysis of randomized clinical trials. JAMA Psychiatry.

[20] Kadriu B, Gold PW, Luckenbaugh DA, Lener MS, Ballard ED, Niciu MJ (2018). Acute ketamine administration corrects abnormal inflammatory bone markers in major depressive disorder. Mol Psychiatry.

[21] Walker AK, Budac DP, Bisulco S, Lee AW, Smith RA, Beenders B (2013). NMDA receptor blockade by ketamine abrogates lipopolysaccharide-induced depressive-like behavior in C57BL/6J mice. Neuropsychopharmacology.

[22] do Vale EM, Xavier CC, Nogueira BG, Campos BC, de Aquino PE, da Costa RO (2016). Antinociceptive and anti-inflammatory effects of ketamine and the relationship to its antidepressant action and GSK3 inhibition. Basic Clin Pharm Toxicol.

[23] Majumder S, Caccamo A, Medina DX, Benavides AD, Javors MA, Kraig E (2012). Lifelong rapamycin administration ameliorates age-dependent cognitive deficits by reducing IL-1beta and enhancing NMDA signaling. Aging Cell.

[24] Gottschalk S, Cummins CL, Leibfritz D, Christians U, Benet LZ, Serkova NJ (2011). Age and sex differences in the effects of the immunosuppressants cyclosporine, sirolimus and everolimus on rat brain metabolism. Neurotoxicology.

[25] Serkova N, Jacobsen W, Niemann CU, Litt L, Benet LZ, Leibfritz D (2001). Sirolimus, but not the structurally related RAD (everolimus), enhances the negative effects of cyclosporine on mitochondrial metabolism in the rat brain. Br J Pharm.

[26] Levy EI, Hanel RA, Tio FO, Garlick DS, Bailey L, Cunningham MR (2006). Safety and pharmacokinetics of sirolimus-eluting stents in the canine cerebral vasculature: 180 day assessment. Neurosurgery.

[27] Thomson AW, Turnquist HR, Raimondi G (2009). Immunoregulatory functions of mTOR inhibition. Nat Rev Immunol.

[28] Cloughesy TF, Yoshimoto K, Nghiemphu P, Brown K, Dang J, Zhu S (2008). Antitumor activity of rapamycin in a Phase I trial for patients with recurrent PTEN-deficient glioblastoma. PLoS Med.

[29] Mahalati K, Kahan BD (2001). Clinical pharmacokinetics of sirolimus. Clin Pharmacokinet.

[30] Berman RM, Cappiello A, Anand A, Oren DA, Heninger GR, Charney DS (2000). Antidepressant effects of ketamine in depressed patients. Biol Psychiatry.

[31] Hawley CJ, Gale TM, Sivakumaran T, Hertfordshire Neuroscience Research g. (2002). Defining remission by cut off score on the MADRS: selecting the optimal value. J Affect Disord.

[32] Zhang Y, Yan H, Xu Z, Yang B, Luo P, He Q (2019). Molecular basis for class side effects associated with PI3K/AKT/mTOR pathway inhibitors. Expert Opin Drug Metab Toxicol.

[33] Abdallah CG, Jackowski A, Salas R, Gupta S, Sato JR, Mao X (2017). The nucleus accumbens and ketamine treatment in major depressive disorder. Neuropsychopharmacology.

[34] Melo A, Kokras N, Dalla C, Ferreira C, Ventura-Silva AP, Sousa N (2015). The positive effect on ketamine as a priming adjuvant in antidepressant treatment. Transl Psychiatry.

[35] Moda-Sava RN, Murdock MH, Parekh PK, Fetcho RN, Huang BS, Huynh TN, et al. Sustained rescue of prefrontal circuit dysfunction by antidepressant-induced spine formation. Science. 2019;364:eaat8078. 10.1126/science.aat8078. 10.1126/science.aat8078 PMC6785189 30975859

[36] Sanacora G (2019). Caution against overinterpreting opiate receptor stimulation as mediating antidepressant effects of ketamine. Am J Psychiatry.

[37] Abdallah CG, Averill LA, Akiki TJ, Raza M, Averill CL, Gomaa H (2019). The neurobiology and pharmacotherapy of posttraumatic stress disorder. Annu Rev Pharm Toxicol.

[38] Gulbins A, Schumacher F, Becker KA, Wilker B, Soddemann M, Boldrin F (2018). Antidepressants act by inducing autophagy controlled by sphingomyelin-ceramide. Mol Psychiatry.

[39] Cleary C, Linde JA, Hiscock KM, Hadas I, Belmaker RH, Agam G (2008). Antidepressive-like effects of rapamycin in animal models: implications for mTOR inhibition as a new target for treatment of affective disorders. Brain Res Bull.

[40] Kara NZ, Flaisher-Grinberg S, Anderson GW, Agam G, Einat H (2018). Mood-stabilizing effects of rapamycin and its analog temsirolimus: relevance to autophagy. Behav Pharmacol.

[41] Diazgranados N, Ibrahim L, Brutsche NE, Newberg A, Kronstein P, Khalife S (2010). A randomized add-on trial of an N-methyl-d-aspartate antagonist in treatment-resistant bipolar depression. Arch Gen Psychiatry.

[42] Zarate CA, Brutsche NE, Ibrahim L, Franco-Chaves J, Diazgranados N, Cravchik A (2012). Replication of ketamine’s antidepressant efficacy in bipolar depression: a randomized controlled add-on trial. Biol Psychiatry.

[43] Nugent AC, Ballard ED, Gould TD, Park LT, Moaddel R, Brutsche NE (2018). Ketamine has distinct electrophysiological and behavioral effects in depressed and healthy subjects. Mol Psychiatry.

[44] Singh JB, Fedgchin M, Daly EJ, De Boer P, Cooper K, Lim P (2016). A double-blind, randomized, placebo-controlled, dose-frequency study of intravenous ketamine in patients with treatment-resistant depression. Am J Psychiatry.

[45] Miller AH, Haroon E, Felger JC (2017). Therapeutic implications of brain-immune interactions: treatment in translation. Neuropsychopharmacology.

[46] Raison CL, Rutherford RE, Woolwine BJ, Shuo C, Schettler P, Drake DF (2013). A randomized controlled trial of the tumor necrosis factor antagonist infliximab for treatment-resistant depression: the role of baseline inflammatory biomarkers. JAMA Psychiatry.

