# Supplementary material for: Modulation of the antidepressant effects of ketamine by the mTORC1 inhibitor rapamycin
Source: Neuropsychopharmacology. 2020 Feb 24;45(6):990–7. doi: 10.1038/s41386-020-0644-9 (PMC7162891; doi:10.1038/s41386-020-0644-9)
Supplement: Supplementary file 1 — Suplemental Information [file 41386_2020_644_MOESM1_ESM.pdf]

SUPPLEMENTAL INFORMATION

CONSORT Flow Diagram

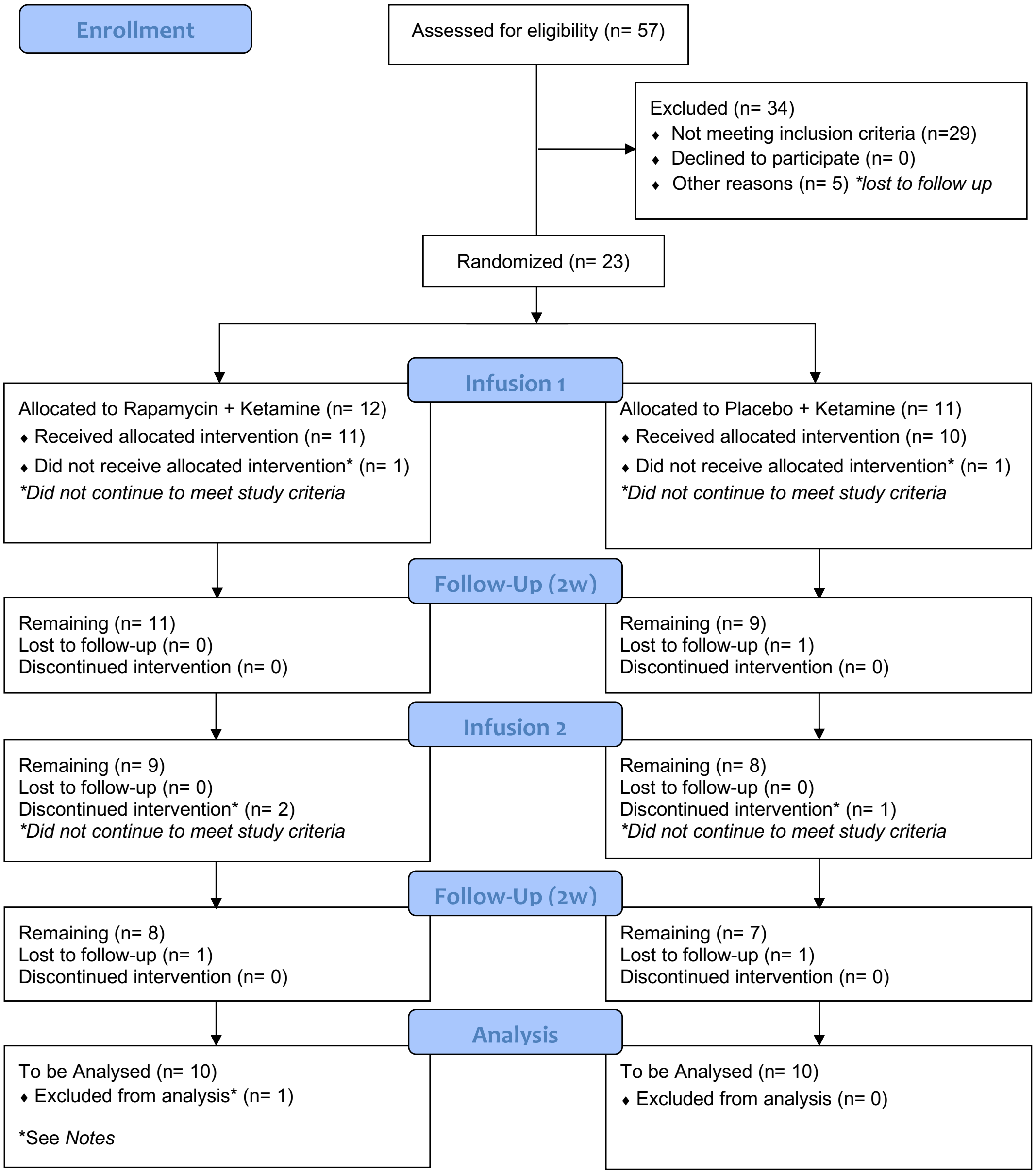

Notes: It was decided a priori to exclude one participant who reported that (s)he received a large dose of hydrocortisone the night prior to the morning randomization. However, for full transparency, a secondary analysis including this participant is conducted and reported.

Additional CONSORT Information

"Discontinued intervention" indicates that the patient did not receive the infusion. Patients "lost to follow-up" are those that we were not able to schedule their follow-up visits. Patients who did "not continue meeting the study criteria" were as follow: At infusion 1, 1 because MADRS scores were less than 18 and 1 because the patient had low oxygen saturation due to sleep apnea. At infusion 2, two patients have MADRS scores less than 18 (1 at the rapamycin and 1 at the placebo sessions) and 1 patient was discontinued per protocol due to worsening of symptoms (at the placebo session).

Additional Analyses

Here we repeated the primary analysis after including the subject who was excluded because of taking a large dose of hydrocortisone the night of treatment day, investigating the effects of the study drug on the primary outcome, Montgomery Åsberg Depression Rating Scale (MADRS). The results were similar to those found in the primary analysis. There was a statistically significant interaction between treatment and time ($F_{(8,261)} = 2.3$, $p = 0.02$). There was also a significant main effect of time ($F_{(8,261)} = 49.0$, $p < 0.0001$). There was no significant main effect of treatment ($F_{(8,261)} = 1.4$, $p = 0.25$).

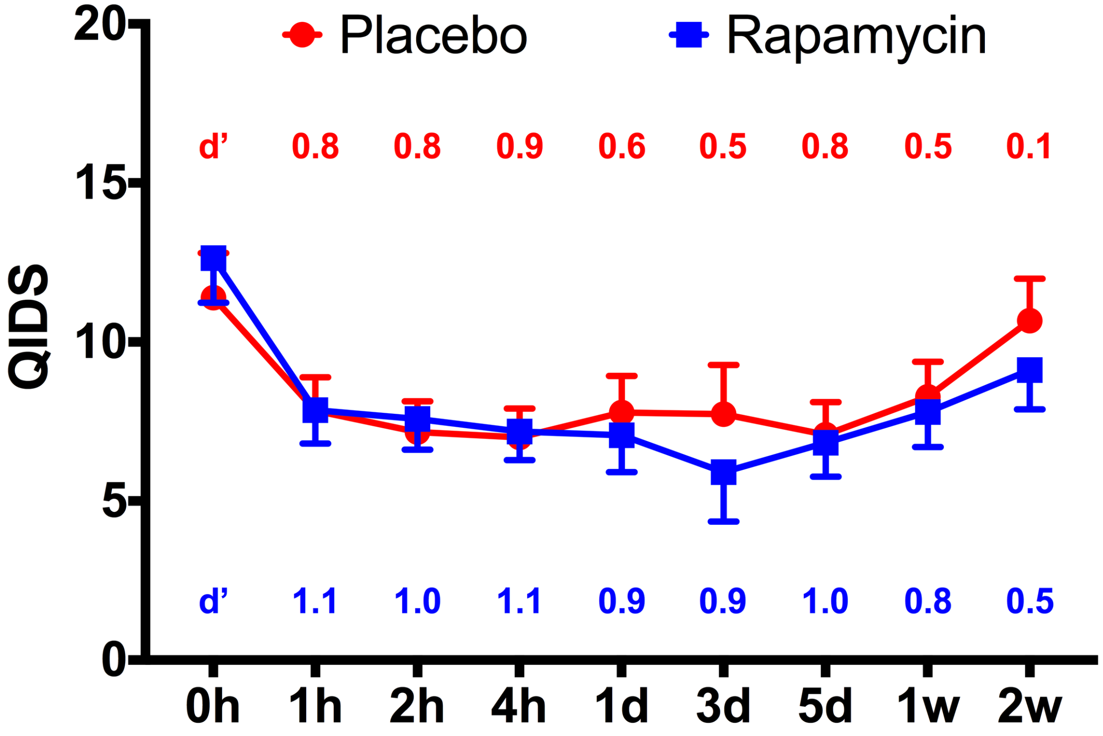

Figure S1. The Study Drug Effect on Quick Inventory of Depressive Symptoms Self-Report (QIDS-SR). There is a significant main effect of time on QIDS-SR ($F_{(8,236)} = 7.1$, $p < 0.0001$), demonstrating significant decrease in QIDS-SR scores from baseline following treatment with either rapamycin+ketamine (Rapamycin; blue line) or placebo+ketamine (Placebo; red line). There is no statistically significant interaction between treatment and time ($F_{(8,236)} = 0.5$, $p = 0.87$). Error bars are standard errors of mean (*SEM*); d` = Cohen’s d` effect size compared to pretreatment QIDS-SR scores;

Table S1. Concomitant Treatments that are prohibited

Use category	Type of medication	Details
**Prohibited**	MAOIs	Prohibited 4-week prior to randomization.
	VNS, ECT, deep brain stimulation	VNS, ECT, or within 6 months at randomization is exclusionary.
	Memantine	Prohibited 4-week prior to randomization.
	Barbiturates	Prohibited 2-week prior to randomization.
	Cidofovir, Mifepristone, Posaconazole, Streptozocin, Ketoconazole, Voriconazole	Prohibited 2-weeks prior to randomization and throughout the study.
	Adenovirus vaccine, live; BCG live intravesical; Influenza nasal vaccine, live; Measles/Mumps/Rubella vaccine, live; Rotavirus vaccine, live; Smallpox vaccine (live vaccinia virus); Typhoid vaccine, live; Varicella vaccine, live; Yellow fever vaccine, live; Zoster vaccine, live	Prohibited 2-weeks prior to randomization and for 3 months after completing the study.
	Strong inducers (e.g., rifampin, rifabutin) and strong inhibitors (e.g., itraconazole, erythromycin, telithromycin, clarithromycin) of CYP3A4 and P-gp	Prohibited 2-weeks prior to randomization and throughout the study.
**Permitted with restrictions***	Benzodiazepines (stable dose)	Benzodiazepines are permitted only if the patient has been taking a stable regimen and dose at least 4 weeks prior to randomization. The dosage prior to randomization should be no more than Clonazepam maximum dose 3 mg/day or equivalent.<br>For patients reporting significant treatment-emergent nervousness, restlessness, and/or akathisia, study clinicians are allowed to increase the dose of the concomitant benzodiazepine anxiolytic up to additional clonazepam 1 mg/day equivalents.
	Modulators of CYP3A4 and P-gp; drugs that could increase sirolimus blood concentrations (e.g., bromocriptine, cimetidine, cisapride, clotrizamole, danzol, diltiazem, fluconazole, protease inhibitors, metoclopramide, nicardipine, troleandomycin, verapamil); drugs and other agents that could decrease sirolimus levels (e.g., carbamazepine, phenobarbital, phenytoin, rifapentine, St. John's Wort); drugs with concentrations that could increase when given sirolimus (e.g., verapamil).	Permitted only at the discretion of the prescriber after careful review of concomitant medications.

Notes: Sedatives, hypnotics, benzodiazepines, sedating antihistamines or other psychotropic medications were not permitted within 8 hours of treatment sessions; except – at the discretion of the investigator – for medications that will results in discontinuation/withdrawal symptoms or that may alter the risk benefit ratio.

Table S2. Averse Events Profile

Adverse Event	Rapamycin	Placebo
Anxiety	1	0
Chest tightness	1	0
Depression	1	1
Diarrhea	1	1
Dizzy	2	0
Drowsy	1	0
Dry mouth	0	1
Fatigue	4	3
Fever	1	0
GI discomfort	2	0
Headaches	3	3
Nausea	3	2
Pain	3	1
Upper Respiratory	2	0
